# Clinical characterizations and molecular genetic study of two co-segregating variants in *PDZD7* and *PDE6C* genes leading simultaneously to non-syndromic hearing loss and achromatopsia

**DOI:** 10.1186/s12920-024-01942-3

**Published:** 2024-07-01

**Authors:** Zahra Nouri, Akram Sarmadi, Sina Narrei, Hamidreza Kianersi, Farzan Kianersi, Mohammad Amin Tabatabaiefar

**Affiliations:** 1 Department of Genetics and Molecular Biology, School of Medicine, Isfahan University of Medical Sciences, Isfahan, Iran; 2 Pediatric Inherited Diseases Research Center, Research Institute for Primordial Prevention of Non-Communicable Disease, Isfahan University of Medical Sciences, Isfahan, Iran; 3 Department of Research and Development, Harmonic Medical Genetics Lab, Isfahan, Iran; 4 Isfahan Eye Research Center, Isfahan University of Medical Sciences, Isfahan, Iran; 5 Department of Ophthalmology, Isfahan University of Medical Sciences, Isfahan, Iran; 6 University of Medical Sciences, Isfahan, 81746-73461 Iran

**Keywords:** Hearing loss, Cone dystrophy, Achromatopsia, Whole exome sequencing, *PDZD7*, *PDE6C*

## Abstract

**Background:**

Autosomal recessive non-syndromic hearing loss (NSHL) and cone dystrophies (CODs) are highly genetically and phenotypically heterogeneous disorders. In this study, we applied the whole exome sequencing (WES) to find the cause of HL and COD in an Iranian consanguineous family with three affected individuals.

**Methods:**

Three members from an Iranian consanguineous family who were suffering from NSHL and visual impairment were ascertained in this study. Comprehensive clinical evaluations and genetic analysis followed by bioinformatic and co-segregation studies were performed to diagnose the cause of these phenotypes. Data were collected from 2020 to 2022.

**Results:**

All cases showed congenital bilateral NSHL, decreased visual acuity, poor color discrimination, photophobia and macular atrophy. Moreover, cornea, iris and anterior vitreous were within normal limit in both eyes, decreased foveal sensitivity, central scotoma and generalized depression of visual field were seen in three cases. WES results showed two variants, a novel null variant (p.Trp548Ter) in the *PDE6C* gene causing COD type 4 (Achromatopsia) and a previously reported variant (p.Ile84Thr) in the *PDZD7* gene causing NSHL. Both variants were found in the *cis* configuration on chromosome 10 with a genetic distance of about 8.3 cM, leading to their co-inheritance. However, two diseases could appear independently in subsequent generations due to crossover during meiosis.

**Conclusions:**

Here, we could successfully determine the etiology of a seemingly complex phenotype in two adjacent genes. We identified a novel variant in the *PDE6C* gene, related to achromatopsia. Interestingly, this variant could cooperatively cause visual disorders: cone dystrophy and cone-rod dystrophy.

## Background

Hearing loss (HL) is a prevalent sensorineural disorder, which is clinically and genetically heterogeneous. Genetic factors account for about 75% of cases, meanwhile the remaining percentage is related to environmental factors and age-relate HL [1]. While there are about 400 types of syndromic HL, about 70–75% of all HL cases are considered to be non-syndromic HL (NSHL), without any further clinical abnormalities. However, most types of NSHL and syndromic HL are caused by pathogenic variants in distinct genes, it is notable that some types of syndromic and non-syndromic forms of HL can be caused by allelic mutations in the same gene [2]. Since many molecular factors are vital for the development and maintenance of the auditory systems, variations in many genes can cause either NSHL or syndromic HL [3]. In Western populations, mutations in the DNFB1 locus, containing *GJB2* and *GJB6* genes, accounts for the etiology of more than 50% of ARNSHL [4]. In Iran congenital HL is the second most frequent disability following intellectual impairment, and the average percentage of *GJB2* mutations is about 18.7% [5, 6].

The PDZ domain-containing 7 (*PDZD7*) gene was identified as the cause of NSHL in a family with a hearing impaired child in 2009 by *Schneider et al.* [7]. The affected boy was recognized to have a homozygous reciprocal translocation, 46,XY, t(10;11) (q24.3;q23.3), with a breakpoint location between exons 10 and 11 out of 17 exons encoding *PDZD7*. Eventually, the *PDZD7* gene, which was mapped to human chromosome 10q24.31, was introduced as DFNB57 (OMIM 618,003). Mutations in this gene in combination with mutations in *ADGRV1*, account for digenic Usher syndrome (OMIM 605,472), the most common syndromic HL, characterized by HL and visual impairment [8]. *PDZD7* encodes a ciliary protein which is highly expressed in inner ear hair cells and is a paralog of harmonin and whirlin proteins [9, 10].

Cone photoreceptor disorders form a clinical spectrum of diseases that include complete and incomplete achromatopsia (ACHM) and progressive cone dystrophy (COD). ACHM is an autosomal recessive condition with clinical presentations beginning from birth or early infancy with poor visual acuity (VA), pendular nystagmus, photophobia, and reduced or absent color discrimination [11]. COD is an advanced cone disorder in which patients originally having normal cone function may show gradually loss of vision during the first or second decade of life [12]. The main characterizations of cone dystrophies are decreased best-corrected visual acuity (BCVA), nystagmus and poor color vision [13, 14].

Phosphodiesterase 6 (PDE6) is an essential component of the phototransduction pathway, which converts light to electrical signals within the neural retina, and is responsible for the regulation of the intracellular concentration of cyclic nucleotides in every tissue. Light activates this enzyme which hydrolyze the intracellular cGMPs, leading to a change in membrane potential and diffusion of the visual cycle [15, 16]. The catalytic alpha subunit of the cone photoreceptor phosphodiesterase, which is the key regulatory component in cone phototransduction, is encoded by the *PDE6C* gene that is mapped on chromosome 10q23.33. Variants in this gene are related to both complete and incomplete ACHM, cone dystrophy, and cone-rod dystrophy (CORD) [17].

Molecular genetic diagnosis is recently undergoing a significant transformation. Next Generation Sequencing (NGS) technology such as targeted NGS (TNGS), whole genome sequencing (WGS) or whole exome sequencing (WES), which are becoming more accurate and cost-effective, offer an opportunity to disease diagnosis and discovery of novel disease-causing genes and variants in highly heterogeneous disorders such as HL [18, 19].

The aim of this study was to identify the molecular pathology of congenital HL and visual impairment in a consanguineous family with three affected girls, using WES.

## Methods

### Subject and clinical evaluations

**Fig. 1:**
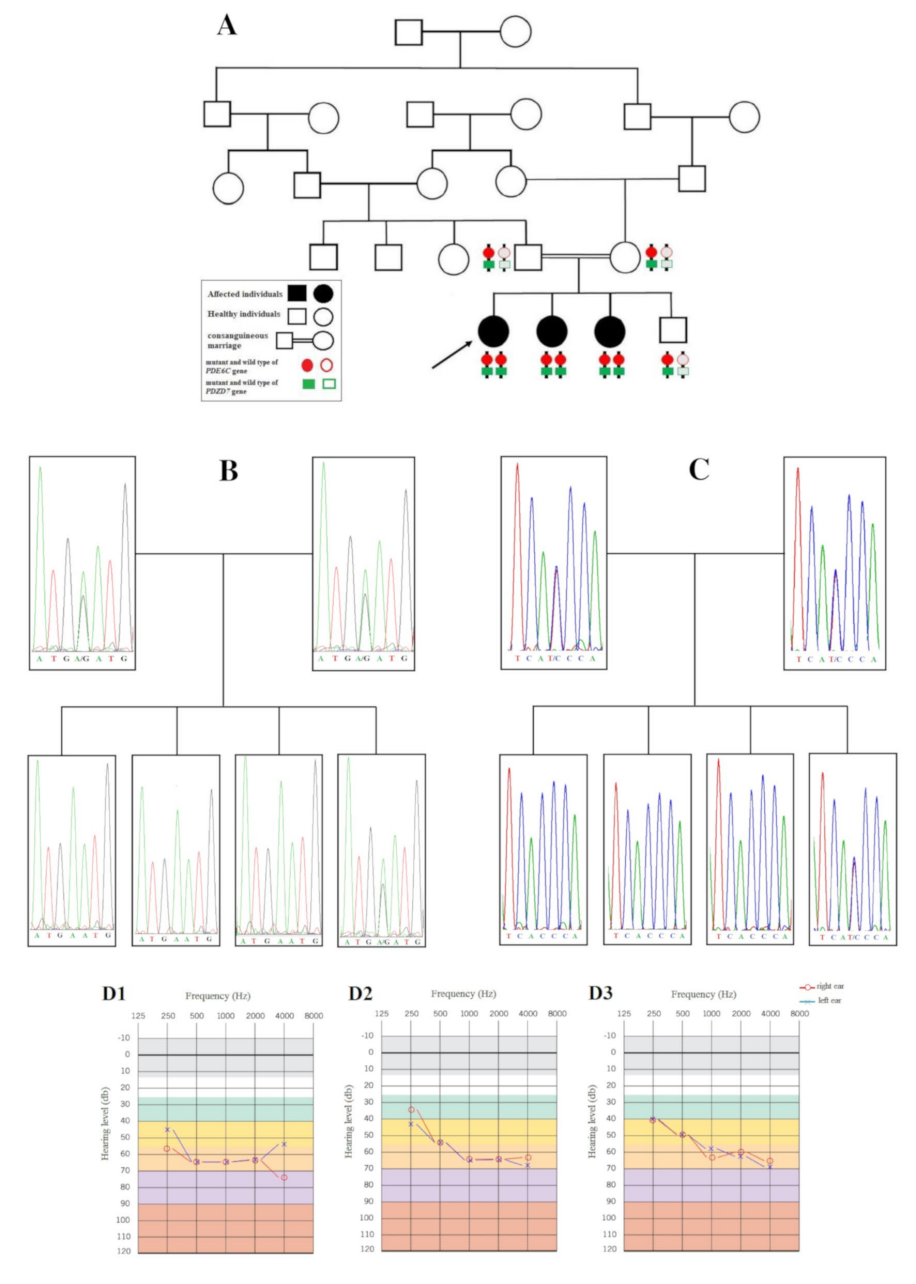
**(A)** The pedigree of the family. The proband who was subjected to WES is marked with an arrow (V-1). The genotype (heterozygous or homozygous) pattern of two variants in both genes (*PDE6C* and *PDZD7*) are schematically shown by red circle and green rectangle. The adjacent variants in affected members are in *cis* configuration. (**B & C**) The electropherograms of the variants. **(B)** co-segregation analysis for *PDE6C* gene variant and **(C)** co-segregation analysis for the *PDZD7* gene variant. Both variants are in heterozygous state in the parents (IV-4 and IV-5) and their healthy son (V-4), but homozygous in three affected girls (V-1, V-2 and V-3). **(D)** Pure tone audiogram of patients (D1, D2 and D3 are for case#1 (V-1), case#2 (V-2) and case#3 (V-3), respectively). Audiogram indicates moderate to severe NSHL in both ears. Frequency in hertz (Hz) and the hearing threshold in decibels (dB) are shown

A forty-one-year-old woman (proband in pedigree, Fig. 1.A) and her two younger sisters (age: 40 and 37 years old) with congenital HL, decreased VA, nystagmus and no color perception from an Iranian consanguineous family were ascertained in this study. They had a younger 32-year-old healthy brother with normal hearing and vision (Fig. 1.A).

Comprehensive family history, audiological testing such as play audiometry, tympanometry, acoustic stapedial reflex, auditory brainstem response (ABR), auditory steady state response (ASSR) and physical examination were done. A variety of noninvasive imaging test for examining retinal structure such as fundus exam, color vision test, spectral-domain optical coherence tomography (SD-OCT) and Fundus autofluorescence (FAF) were also performed after the measurement of VA, visual field and the intraocular pressure (IOP).

Written informed consent was signed by all the family members through interviews. The study was approved by the Review Board of Isfahan University of Medical Sciences (grant no: 2,400,173 and ethics code: IR.ARI.MUI.REC.1400.011).

### Molecular study

#### DNA extraction, GJB2 sequencing

Genomic DNA was extracted from the peripheral blood of the proband (V-1 in the pedigree) using Prime Prep Genomic DNA Extraction kit from blood (GeNet Bio, South Korea) according to the manufacturer’s instruction. After that Sanger sequencing was performed in order to exclude mutations in exon 2 of the *GJB2* as the most common deafness gene (Table 1.A) [20].

**Table 1:** Primer sequences and in silico analysis of the identified variants

A) Primer sequences using for *GJB2*, *PDE6C* and *PDZD7* genes in Sanger sequencing											
Gene	Exon number	Forward primer (5´ to 3´)				Reverse primer (5´ to 3´)					
*GJB2*	2	CTCCCTGTTCTGTCCTAGCT				CTCATCCCTCTCATGCTGTC					
*PDE6C*	13	CTGTGGCTCTGCTTCACTGA				GACTCATCTGCAGCGGAGAG					
*PDZD7*	3	CAACCTAGCCTTTGCAGGC				CACTGCTGCCTTCCTCCAC					
**B) ** ***In silico *** **analysis of identified variants**											
Gene	Genomic location (hg19/GRCh37)	Exon number	cDNA change	amino acid change	1000 Genome MAF	ExAC MAF	CAAD score	Mutation Taster	PROVEAN	PolyPhen	ACMG Classification
*PDZD7*	Chr10: 102,783,801	3	c.T251C	p. Ile84Thr	NA	NA	25.5	Disease causing	Damaging	Possibly damaging	PM2, PP2, PP3, PP4, PP5 **Likely pathogenic**
*PDE6C*	Chr10: 95,400,221	13	c.G1644A	p.Trp548Ter	NA	NA	44	Disease causing	-	-	PVS1, PM2, PP3 **Pathogenic**

MAF: minor allele frequency, NA: not available, PM: pathogenic moderate, PP: pathogenic supporting, PVS: pathogenic very strong

#### WES and bioinformatics analyses

About 300 ng of genomic DNA from the proband (V-1) was prepared to carry out WES. The Sample was sent to Macrogen (South Korea) and was subjected to WES using the NovaSeq 6000 platform (Illumina, US). The mean depth of coverage was 150X for greater than 92% of targeted regions. The clean reads were aligned to the reference human genome sequence (hg19) using the Burrows-Wheeler Aligner (BWA) (http://bio-bwa.sourceforge.net/) and then the GATK Software (https://www.broadinstitute.org/gatk/) was used for base quality re-calibration and variants calling. All the steps of this technique were the same as we explained in our previous articles [19, 21]. The potential pathogenicity of variants was assessed using online prediction software tools, such as PolyPhen-2, MutationTaster, PROVEAN and CADD score. The pathogenicity of founded variants were classified based on the American College of Medical Genetics and Genomics (ACMG) guidelines [22].

#### Variant confirmation

All putative disease associated variants were validated and tested for segregation with the phenotype (HL and color vision defect) in all family members by conventional Sanger sequencing. PCR amplification and sequencing of the fragments were performed using the primers listed in Table 1.A.

Electropherograms were compared with reference sequence using SeqMan software version 5.00 © (DNASTAR, Madison, WI, USA). Next, the novelty of the variant or its association with HL, vision impairment and color blindness were investigated in the Human Gene Mutation Database (HGMD), LOVD and the literature.

### The 3D structure of PDZD7 and PDE6C

The *PDZD7* gene is composed of 17 exons encoding a protein with 1033 residues, which folds in four domains. The PDE6C protein consists of three domains and contains 858 amino acids. We used Swiss-Model (https://swissmodel.expasy.org/) and AlphaFold (https://alphafold.ebi.ac.uk) servers to construct the 3D structure of both proteins. The 3D structures were visualized and analyzed with UCSF Chimera (https://www.cgl.ucsf.edu/chimera/).

## Results

### Clinical findings

All three daughters in this family had congenital bilateral NSHL revealed by audiological reports and clinical manifestations. History and physical examination of patients did not reveal any environmental factor as a cause of HL and suggested non-syndromic form of HL. Color blindness, day light sensitivity and photophobia were seen from childhood in all three sisters, and their VA was decreased gradually and progressively.

Clinical presentations, ophthalmic history and the last visual outcomes are described in more detail below:

#### Case #1 (V-1 in the pedigree)

A 41-year-old woman showed congenital bilateral moderate to severe NSHL, according to the audiological evaluations (Fig. 1.D1). Decreased VA, photophobia and light sensitivity were evident since childhood. At the age of 6 years, the lack of color discrimination was diagnosed by her teacher. Progressive loss of VA and high myopia were noticeable during adulthood until she gradually developed night blindness. On the recent examination, her VA was 3 m finger Count (FC) in her right eye (OD) and 4 m FC in her left eye (OS). IOP was 15 mm Hg OD and 16 mm Hg OS and spherical equivalent of refractive error was − 8.5 diopters and − 8 diopters OD and OS, respectively.

In Slit lamp examination (SLE) we found that Cornea, Iris, Lens and anterior vitreous were within normal limit in both eyes. In fundus exam, fine mottling, decreased macular reflex was present. Also, pathologic myopia with tilted disc was accompanied by mild temporal optic atrophy and parapapillary atrophy of RPE and choroid, scleral crescent and a blond fundus, allowing visualization of choroidal vessels. Color vision test showed no color perception in both eyes. SD-OCT result showed macular tinning and decreased macular volume, accompanied by loss of the outer retinal layers affecting the macula. FAF revealed Focally increased AF at the macula; perifoveal rings of increased autofluorescence surrounded by a rim of decreased AF; “bull’s-eye” appearance. The photoreceptor layer is destroyed while the rod layer is still remaining, thus, the final diagnosis for her is Cone dystrophy.

#### Case #2 (V-2 in the pedigree)

A 40-year-old woman had congenital bilateral moderate to severe NSHL, according to the audiological evaluations (Fig. 1.D2). Poor eyesight, photophobia and lack of color discrimination were her obvious features since childhood. Since childhood, progressive loss of VA in day light, photophobia and lack of color discrimination were reported. On examination, her VA was 2 m FC in her OD and 3 m FC in her OS. IOP was 18 mm Hg OD and 19 mm Hg OS and spherical equivalent of refractive error was − 4 diopters and − 4 diopters OD and OS, respectively.

Cornea, iris and anterior vitreous were within normal limit in both eyes in the result of SLE and 2 + posterior sub capsular cataract (PSCC) was evident in both eyes. In funduscopy imaging, mild pigmentary changes in macula and 2 + optic disc pallor, which is more prominent in the temporal side of the disc, were seen in both eyes. Color vision test result showed no color perception in both eyes. Macular tinning and decreased macular volume, accompanied by diffuse tinning of photoreceptor layer and loss of photoreceptor layer in fovea, was shown in SD-OCT. As the result of FAF, focal increased autofluorescence at the macula and spots of increased and decreased autofluorescence around it and a small spot of hypo- autofluorescence was seen in the center of fovea. She was diagnosed as Cone-Rod dystrophy due to complete destruction of the photoreceptor layer.

#### Case #3 (V-3 in the pedigree)

A 37-year-old woman with congenital bilateral moderate to severe NSHL, according to the audiological evaluations (Fig. 1.D3). She had Poor eyesight, photophobia and lack of color discrimination. Loss of VA was progressive specially in day light and she also eventually developed night blindness. On examination, her VA was 4 m finger Count (FC) in her right eye (OD) and 4 m FC in her left eye (OS). IOP was 17 mm Hg OD and 17 mm Hg OS and spherical equivalent of refractive error was − 4.5 diopters and − 4 diopters OD and OS, respectively.

SLE result exactly resembled that of her eldest sister and in funduscopy imaging a symmetric pigmentary changes, and bull’s-eye pattern of macular atrophy with moderate temporal optic atrophy were seen and peripheral fundus appeared to be normal. She also had no color perception in both eyes upon color vision test. The SD-OCT test showed severe macular tinning and decreased macular volume in both eyes. In macular region, disruption of all retinal layers in the right eye and the outer retinal layers in the left eye, accompanied by disorganization of RPE and choriocapillaris, was present. As the result of FAF, a dense, round hypo-AF at the center of the macula with a rim of hyper-AF and a ring of reduced AF around it was seen in both eyes. The final diagnosis for this patient is Cone dystrophy like as her eldest sister.

**Fig. 2:**
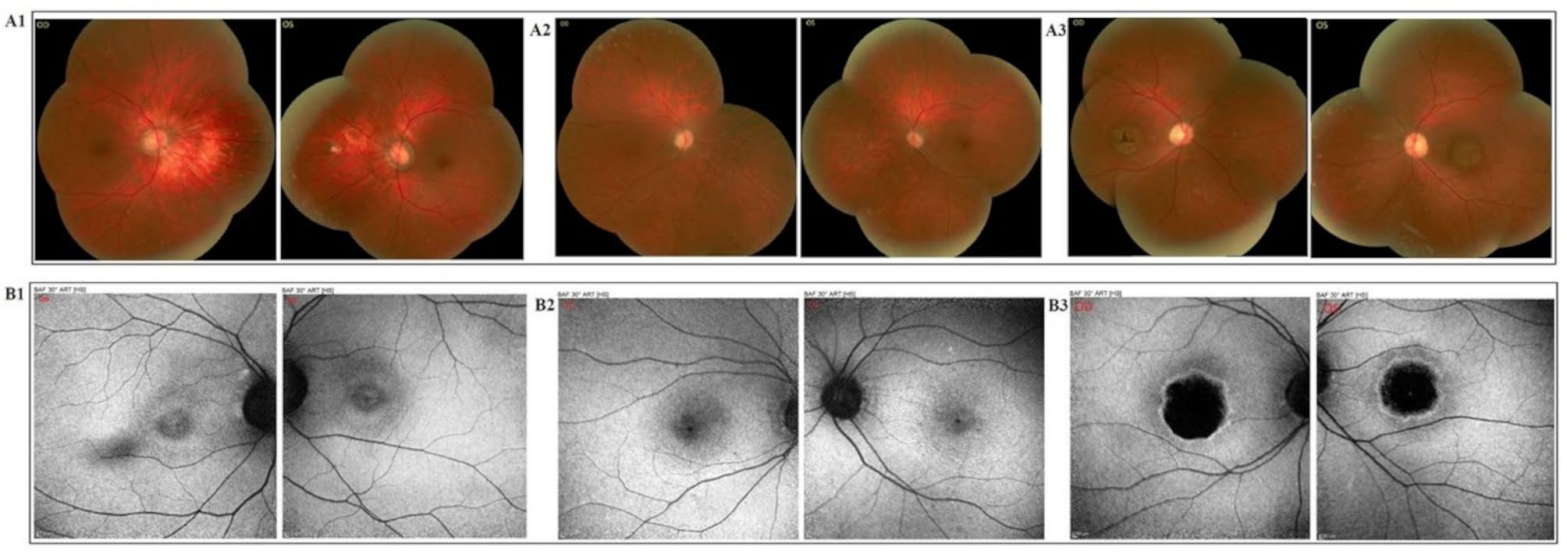
**A1-3)** Funduscopy of our three patients (**A1**: Fine mottling, decreased macular reflex, pathologic myopia with tilted disc accompanied by mild temporal optic atrophy, parapapillary atrophy of RPE and choroid, scleral crescent and a blond fundus are seen. **A2**: There is mild pigmentary changes in macula and 2 + optic disc pallor which is more prominent in temporal side of the disc. **A3**: A symmetric pigmentary changes and bull’s-eye pattern of macular atrophy with moderate temporal optic atrophy are seen and peripheral fundus appears normal). **B1-3**: Fundus autofluorescence (FAF) of our three patients (**B1**: There is focal increased AF at the macula; perifoveal rings of increased autofluorescence surrounded by a rim of decreased AF with a “bull’s-eye appearance. **B2**: Focal increased autofluorescence at the macula, spots of increased and decreased autofluorescence around it and a small spot of hypo- autofluorescence are seen in the center of fovea. **B3**: There is a dense, round hypo-AF at the center of the macula with a rim of hyper-AF and a ring of reduced AF around it)

**Fig. 3:**
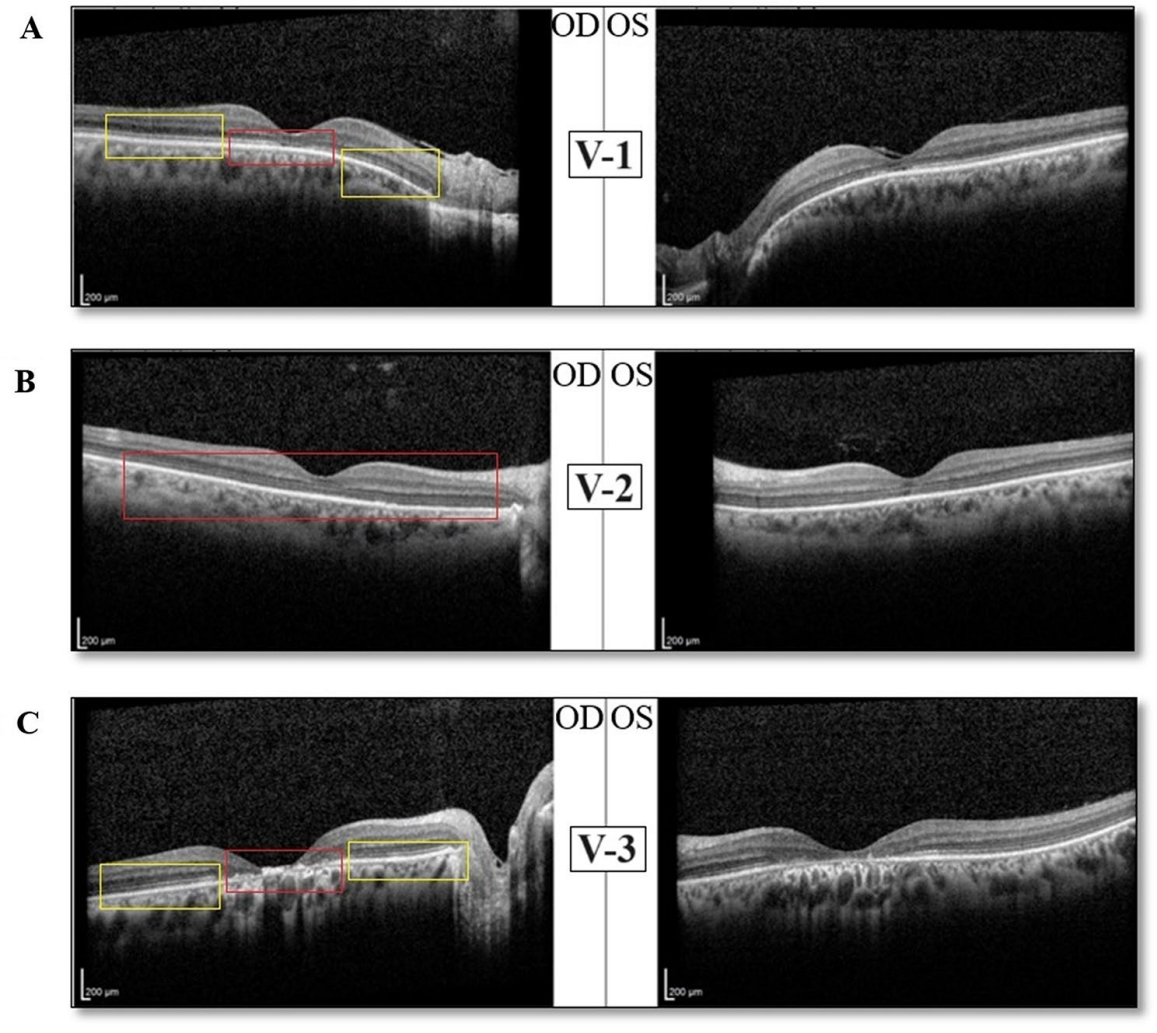
(**A, B & C**) Spectral-domain optical coherence tomography (SD-OCT) imaging of our three cases. **A**: The OCT image shows macular tinning and decreased macular volume accompanied by loss of the outer retinal layers affecting the macula and a fine epiretinal membrane is seen on the macula of the left eye. The red box indicates the complete destruction of the cone cells and the yellow box shows that the Rod cell layers are relatively natural. Thus, Cone dystrophy is diagnosed for this patient. **B**: Macular tinning and decreased macular volume accompanied by diffuse tinning of photoreceptor layer and loss of photoreceptor layer in fovea are seen. Because of the absent of photoreceptor layer in this patient, the diagnosis is Cone-rod dystrophy. **C**: in this patient SD-OCT shows sever macular tinning and decreased macular volume in both eyes. In the macular region, disruption of all retinal layers in the right eye and the outer retinal layers in the left eye is accompanied by disorganization of RPE and choriocapillaris is present. Also, attenuation and tinning of photoreceptor layer in extrafoveal region in both eye is seen. Just like patient #1, the Cone cells are completely destroyed but the Rod layer are relatively normal, Thus, for this patient the diagnosis is Cone dystrophy, too

Electroretinography (ERG) results were not available for any of the patients, but the results of other tests revealed the exact diagnosis for each one. Funduscopy and FAF imaging of three cases are shown in Fig. 2 and SD-OCT results are shown in Fig. 3.

### Molecular findings

Direct sequencing of the coding exon of the *GJB2* gene did not show any mutation. WES was applied and totally 26,961 variants were detected. After using our pipeline and filtering strategy (Table 2), two variants in two different genes met the criteria for further analyses. The two pathogenic identified variants were a homozygous variant in the *PDZD7* gene (NM_001195263.2), c.251T > C (p.Ile84Thr), which was previously reported as a disease causing mutation in an Iranian family [23], and a homozygous novel stop gain variant, c.1644G > A (p.Trp548Ter), in the *PDE6C* gene (NM_006204.4). This novel variant was predicted to be deleterious by MutationTaster as well as several other prediction tools (Table 1.B) and were also absent from dbSNP version 147, 1000 genomes project phase 3, NHLBI GO ESP, ExAC, Iranome, HGMD, GTaC and Clinvar databases and were not found in the literature, either.

**Table 2:** Filtering strategy in this research to identify the causative variants and the number of variant which were filtered in each step

Total number of variants	26,961
Exonic and splicing variants	**23,467**
Remove synonymous variants	**11,620**
Homozygous/ Compound heterozygous	**4594**
Functional variants with MAF < 0.01	**62**
Variants matching with the phenotype of the patients and segregate within family	***PDZD7***: **(p.Ile84Thr)** ***&*** ***PDE6C***: **(p.Trp548Ter)**

### Variant confirmation and co-segregation analysis

The variants were found to be co-segregating with the two phenotypes in the family: father and mother were heterozygous for both variants, the three sisters were homozygous and their only healthy brother was heterozygous. DNA sequence electropherogram of the family members are shown in Fig. 1.B & C.

According to the ACMG guidelines, the variant in the *PDE6C* and *PDZD7* genes could be classified as pathogenic and likely pathogenic variants met the PVS1, PM2, PP3 and PM2, PP2, PP3, PP4, PP5 criteria, respectively.

### The 3D structure of PDZD7 and PDE6C

**Fig. 4:**
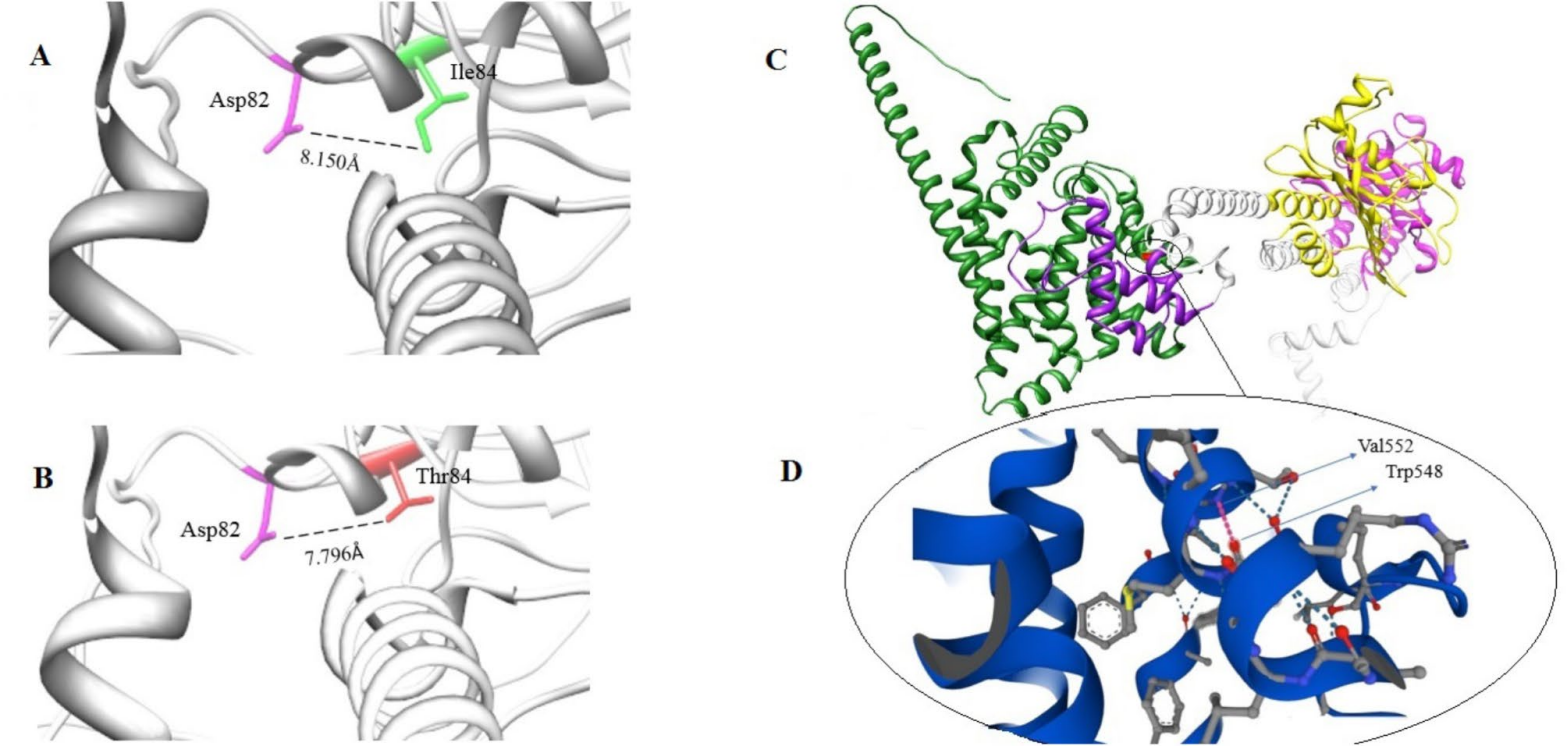
The 3D structure of the mutated region of the PDZD7 (**A** & **B**) and all domains of PDE6C (**C** & **D**). **(A)** Ile84 is bond to Asp82 with a distance of 8.150Å. **(B)** In the mutated protein, substitution of Ile with Thr makes the distance shorter to 7.796Å. **(C)** Three functional domains of PDE6C protein, GAF1 (amino acids 75–224), GAF2 (amino acids 256–433) and PDEase domain (amino acids 486–819). GAF1 and GAF2 are shown with pink and yellow color, respectively. The PDEase domain is shown by green (amino acids 486–548) and purple (amino acids 549–819 which are deleted as the result of the null variant). **(D)** Hydrogen bond between Trp548 and Val552 is shown by red spots, disrupted by substitution of Trp by the stop codon

In the wild-type of the PDZD7 protein, isoleucine at position 84 had a hydrogen bond with aspartic acid at position 82 with a distance of 8.150Å. While, in the mutated protein, in which isoleucine is replaced by threonine, the distance had become shorter to 7.796Å (Fig. 4A-B). To assess the protein stability of this modification, the mCSM (http://structure.bioc.cam.ac.uk/mcsm) was used to calculate ΔΔ*G* and it was showed that the variant resulted in destabilization of the mutated protein (ΔΔ*G* = 0.52 kcal/mol). The p.Trp548Ter variant in the *PDE6C* gene is located in the PDEase domain of the protein (three domains, GAF1, GAF2 and PDEase, of the PDE6C protein are shown in Fig. 4C). As a result of this variant, tryptophan at position 548 which is bond to valine at residue 552 is changed to stop codon (Fig. 4.D). This hydrogen bond in the PDEase domain plays a critical role in maintenance of the protein stability.

## Discussion

Hearing loss (HL) is the most frequent sensory disorder with more than 90 genes identified causing Non-syndromic HL (NSHL). The *PDZD7* gene was firstly recognized in 2009 by *Schneider et al.* as a gene related to NSHL [7]. This gene encodes a protein containing four domains, three PDZ and one HHD (harmonin homology domain). While the PDZ3 only interacts with WHRN, the other two PDZ domains have important role in interacting with other Usher quaternary protein complex components USH2A, ADGRV1 and WHRN [24]. This interaction and the proper function of these proteins are essential for development and correct performance of both auditory and visual systems [25]. To date, 31 different pathogenic variants have been reported in the *PDZD7* gene, causing ARNSHL, RP or usher syndrome type 2 (USH2) [8, 23, 26, 27]. The p.Ile84Thr variant in the *PDZD7* gene which was found to be co-segregating in our patients, is a missense variant in the PDZ1 domain. The substitution of isoleucine with threonine as a polar amino acid may disrupt the hydrogen bond, decreasing the interaction of other residues and resulting in destabilizing of the protein. This variant was previously reported in a patient with NSHL without any symptoms of visual or retinal disorder [23]. Up to now, only two other pathogenic variants have been reported in this critical domain, p.Gly103Arg and p.Arg164Trp, which were reported in an Iranian and a Korean NSHL patient with no definite abnormalities in their ophthalmologic examinations, respectively [28, 29].

Cone dystrophies (CODs) are described with poor visual acuity (VA), photophobia, absent or reduced color discrimination and nystagmus with an autosomal recessive pattern of inheritance [30]. Pathogenic variants in different genes such as *CNGA3*, *CNGB3*, *GNAT2*, *PDE6H*, *ATF6* and *PDE6C* have been reported to be relevant to COD, cone-rod dystrophy (CORD) and achromatopsia (ACHM) diseases [31–36]. Mutations in *CNGA3* and *CNGB3* genes are more frequent while pathogenic variants in *PDE6C* are less common. The clinical features are variable in patients with pathogenic variant in different gene [37, 38]. For instance, funduscopy imaging in COD/CORD patients shows a ranges of macular appearance, from normal to bull’s-eye maculopathy to even more severe macular atrophy with feasible pigmentary changes in the periphery in CORD cases [39]. It is notable that, macular atrophy is a rare feature in PDE6C patients, while it is more commonly seen in patients with pathogenic variants in other genes especially *CNGB3* [40, 41]. The PDE6C patients usually have normal fundus examination and minor macular changes. However, in addition to decreased VA and nystagmus, complete ACHM, macular atrophy and COD/CORD are also evident in our patients due to the novel pathogenic variant in the *PDE6C* gene. case 1 and 3, who were diagnosed as COD, had bull’s pattern of maculopathy. While, mild pigmentary changes in macula were seen in the second case with a diagnosis of CORD. The progressive degeneration of the cone and even the rod structure which was obvious in our patients, is a proven feature among patients with pathogenic variants in *CNGA3*, *CNGB3*, and *PDE6C* genes [30, 42, 43]. Decrease in outer nuclear layer and central macular thicknesses, in addition to novel or enlarging disruption of the inner segment ellipsoid line are common clinical presentations among patients with ACHM, which were also seen in our patients [42].

PDE6 catalytic core in rods is a heterodimer of PDE6A and PDE6B, whereas, in the cones the catalytic unit is a homodimer of PDE6C [44]. Different variants in the *PDE6C* gene may result in improperly folded or truncated proteins that could restrict its transport toward the outer segments of the rods by the loss of function mechanism [45]. The novel null pathogenic variant, p.Trp548Ter in the *PDE6C* gene, is located in the PDEase domain, which has an important role in regulating cAMP catabolism and is a crucial step in signal transduction pathway [45]. As the result of this substitution, the hydrogen bond between tryptophan 548 and valine 552 is disrupted and may cause the formation of a non-functional protein. Another possibility is that the produced mRNA will be subjected to nonsense mediated decay (NMD) pathway. Another variant in this position, p.W548L, was reported previously to cause COD without macular dystrophy [46]. While, the p.Trp548Ter variant causes COD/CORD in addition to macular atrophy in our patients. Thus, this variant has caused two different phenotypes in a family. Similarly, four other pathogenic variants in the *PDE6C* gene were also reported, which simultaneously cause COD/CORD [47].

In this study, genetic analysis was performed in three patients with a complex phenotype. Initially, the possibility of a syndromic disorder was considered. However, since we found double homozygous pathogenic variants in two adjacent genes on the same chromosome leading to these two distinct phenotypes, this hypothesis was ruled out. Because both genes are located on chromosome 10q2 (*PDZD7* on 10q23 and *PDE6C* on 10q24) with an average distance of 8.3 centimorgan (cM), based on the deCODE Icelandic project [48], we assumed both variants should be co-segregating with the phenotypes in the family. Upon genotyping the family members, we found that this was confirmed and it was shown that the variants were positioned in the *cis* configuration. Since the possibility of occurring crossover between these two adjacent variants is about 8.3% in each meiosis of the affected patients, it is possible that the two diseases appear independently in subsequent generations. In this regard, genetic counseling can be very helpful to make people aware of the likelihood of disease transmission to the next offspring.

## Conclusions

Here, we showed that a complex phenotype resulting from co-segregation of two genes, could successfully resolved via WES and the following variant analysis. Clinical evaluation and genotype-phenotype correlation were performed, which finally identified two pathogenic variants, one of which was novel, in two distinct but adjacent genes as the cause of the phenotypes. To the best of our knowledge, this is the first report of two alterations in two different genes co-segregating together in an Iranian family. WES is recommended as a powerful method to facilitate identifying potentially causative variants in case of diseases with complex phenotypes, which should be considered that this phenotype may be results from more than one variant in more than one gene. Additionally, these findings contribute to more efficient genetic counselling and the following prevention in the subsequent generations.

## Data Availability

The raw datasets generated and/or analyzed during the current study are not publicly available because it is possible that individual privacy could be compromised, but are available from the corresponding author on reasonable request.
