# Correction: Clinical characterizations and molecular genetic study of two co-segregating variants in PDZD7 and PDE6C genes leading simultaneously to non-syndromic hearing loss and achromatopsia

**DOI:** 10.1186/s12920-025-02197-2

**Published:** 2025-08-04

**Authors:** Zahra Nouri, Akram Sarmadi, Sina Narrei, Hamidreza Kianersi, Farzan Kianersi, Mohammad Amin Tabatabaiefar

**Affiliations:** 1 Department of Genetics and Molecular Biology, School of Medicine, Isfahan University of Medical Sciences, Isfahan, Iran; 2 Pediatric Inherited Diseases Research Center, Research Institute for Primordial Prevention of Non-Communicable Disease, Isfahan University of Medical Sciences, Isfahan, Iran; 3 Department of Research and Development, Harmonic Medical Genetics Lab, Isfahan, Iran; 4 Isfahan Eye Research Center, Isfahan University of Medical Sciences, Isfahan, Iran; 5 Department of Ophthalmology, Isfahan University of Medical Sciences, Isfahan, Iran; 6 University of Medical Sciences, Isfahan, 81746-73461 Iran

**Correction: Nouri et al. BMC Medical Genomics 17, 173 (2024)**

**https://doi.org/10.1186/s12920-024-01942-3**

Following the publication of the original article, a reader reported that in Fig. 1, the panels in section B for individuals IV-4 and IV-5 appeared to be identical. The authors clarified that regarding Fig. 1b of the article, which is suspected to be identical, the image is related to the sequence of the parents of three affected patients with non-syndromic hearing loss and achromatopsia, who are homozygous for two variants in the PDEC6 and PDZD7 genes simultaneously.

The members of this family have been thoroughly investigated in this study, and there is no doubt about the heterozygous nature of the parents due to the homozygous nature of the patient for the variant and the consanguineous marriage of the parents. The authors checked the Sanger sequencing files of the region in the PDEC6 gene of the parents and noticed that the sequences of them (both heterozygous) are so identical, which is consistent with scientific logic.

However, by paying more attention to the noise of the sequence in the parents, the authors found that it is very similar in both sequences, raising the suspicion that both images are the same. It is possible that when drawing the figure, the same sequence of one of the parents was inadvertently inserted. The authors checked the original electropherograms of both parents and found extraordinary similarity in terms of their noise in the favorable region. Since they had some differences in other regions, one can identify them as different. The authors had filed another file for the related parent and considered that for redrawing the image.

Regarding Fig. 2, concerning the funduscopy images, the authors believed that they must have mistakenly missed the images of one of the patients and included the images of one single patient for two patients. These figures are now revised and attached here.

**Incorrect Figures**

**Figure 1**


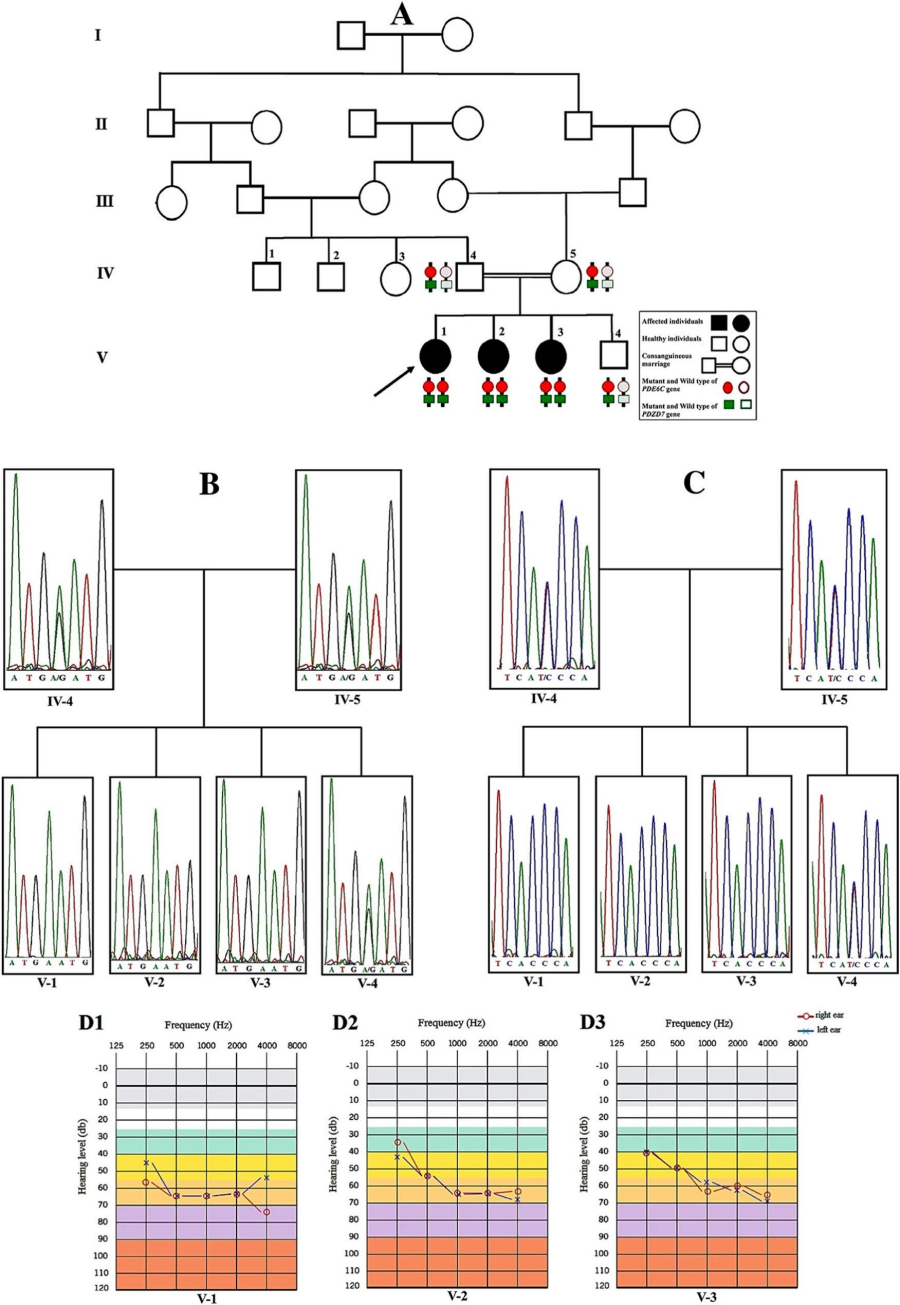


**Figure 2**


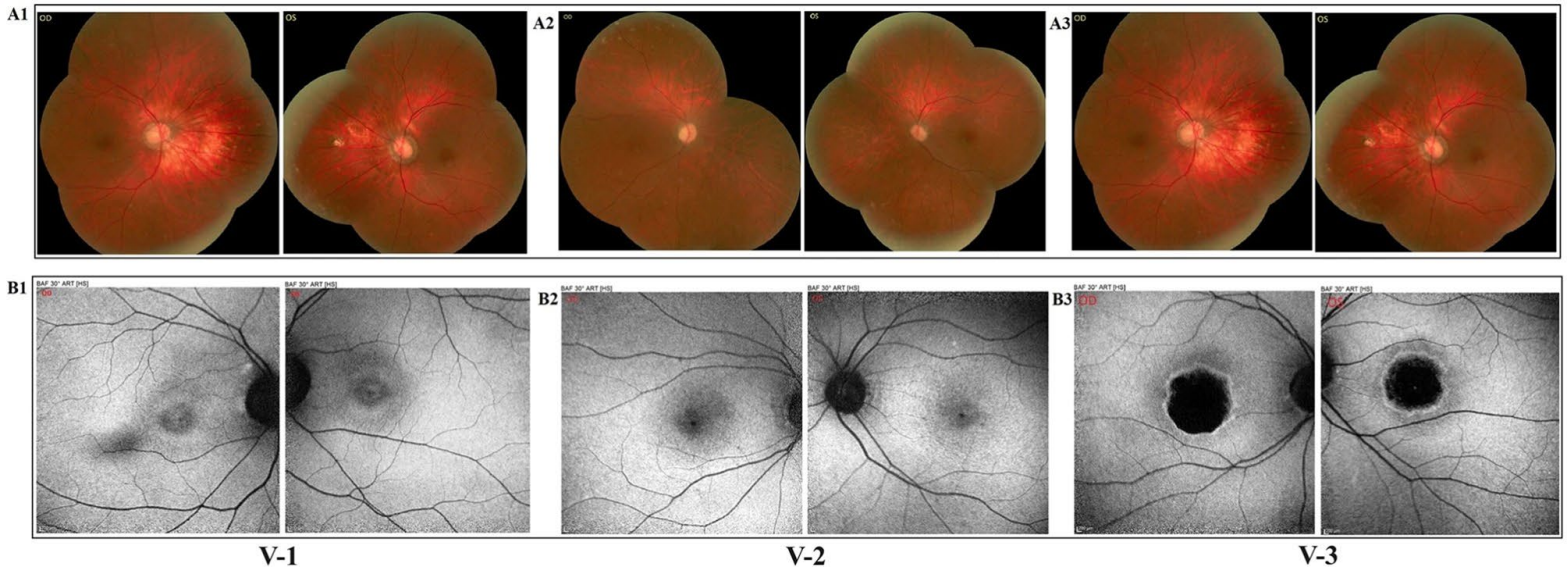


**Correct Figures**

**Fig. 1:**
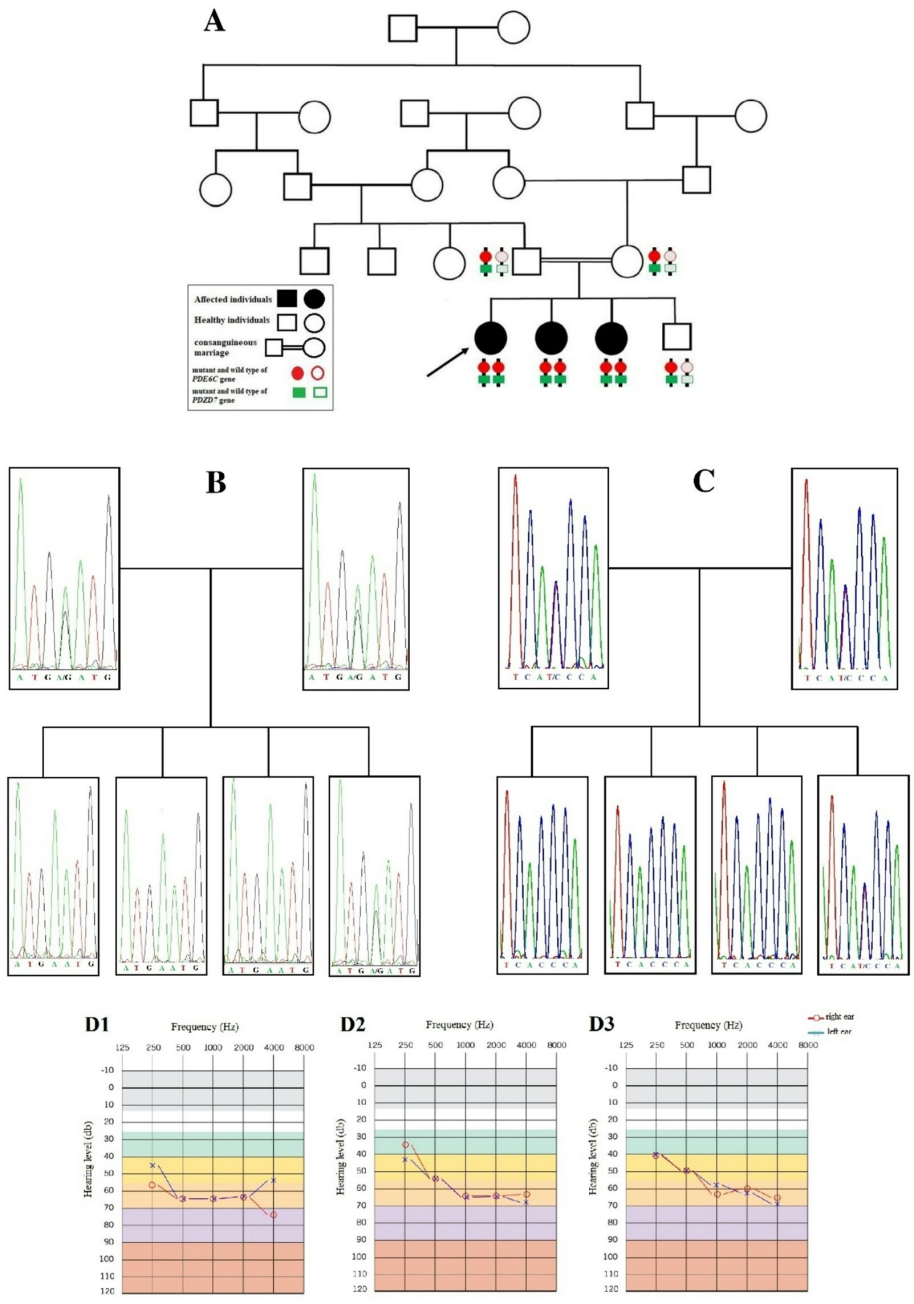
**A** The pedigree of the family. The proband who was subjected to WES is marked with an arrow (V-1). The genotype (heterozygous or homozygous) pattern of two variants in both genes (*PDE6C* and *PDZD7*) are schematically shown by red circle and green rectangle. The adjacent variants in affected members are in *cis* configuration. **B **&** C** The electropherograms of the variants. **B** co-segregation analysis for *PDE6C* gene variant and (**C**) co-segregation analysis for the *PDZD7* gene variant. Both variants are in heterozygous state in the parents (IV-4 and IV-5) and their healthy son (V-4), but homozygous in three affected girls (V-1, V-2 and V-3). **D** Pure tone audiogram of patients (D1, D2 and D3 are for case#1 (V-1), case#2 (V-2) and case#3 (V-3), respectively). Audiogram indicates moderate to severe NSHL in both ears. Frequency in hertz (Hz) and the hearing threshold in decibels (dB) are shown

**Fig. 2:**
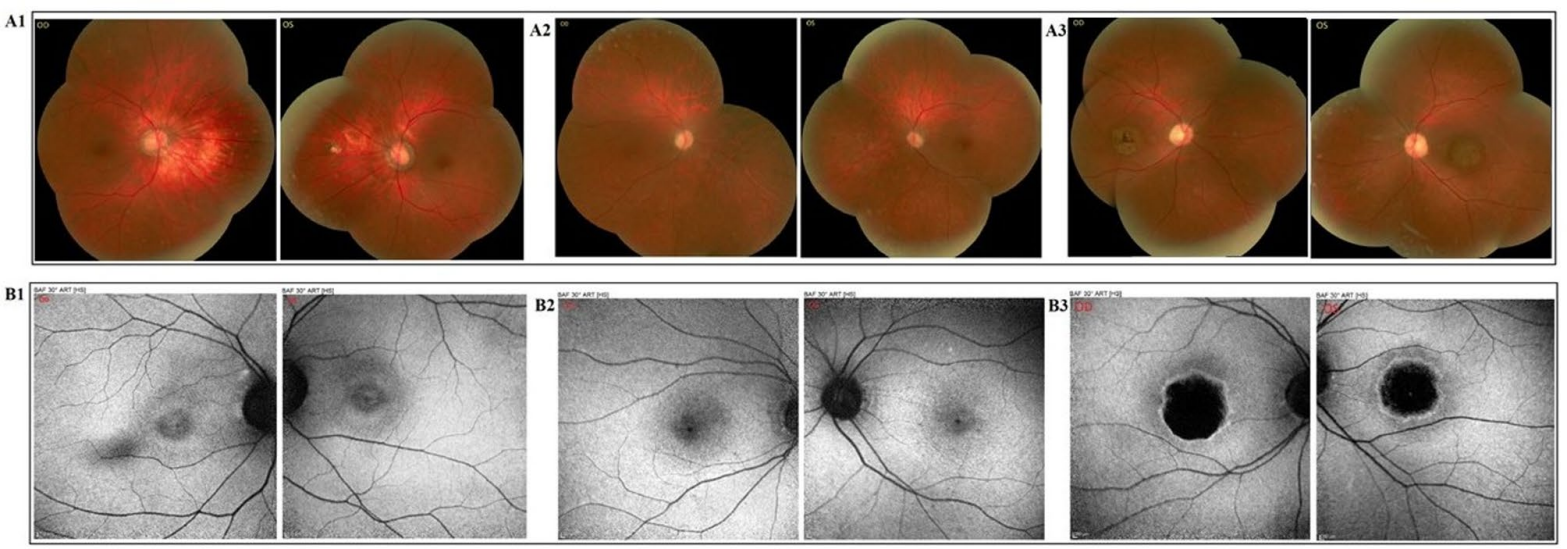
**A1-3** Funduscopy of our three patients **A1** Fine mottling, decreased macular reflex, pathologic myopia with tilted disc accompanied by mild temporal optic atrophy, parapapillary atrophy of RPE and choroid, scleral crescent and a blond fundus are seen. **A2** There is mild pigmentary changes in macula and 2 + optic disc pallor which is more prominent in temporal side of the disc. **A3** A symmetric pigmentary changes and bull’s-eye pattern of macular atrophy with moderate temporal optic atrophy are seen and peripheral fundus appears normal). **B1-3** Fundus autofluorescence (FAF) of our three patients **B1**: There is focal increased AF at the macula; perifoveal rings of increased autofluorescence surrounded by a rim of decreased AF with a “bull’s-eye appearance. **B2** Focal increased autofluorescence at the macula, spots of increased and decreased autofluorescence around it and a small spot of hypo- autofluorescence are seen in the center of fovea. **B3** There is a dense, round hypo-AF at the center of the macula with a rim of hyper-AF and a ring of reduced AF around it)

The original article has been updated accordingly.

